# The whole-organism heavy chain B cell repertoire from Zebrafish self-organizes into distinct network features

**DOI:** 10.1186/1752-0509-5-27

**Published:** 2011-02-10

**Authors:** Rotem Ben-Hamo, Sol Efroni

**Affiliations:** 1 The Mina and Everard Goodman Faculty of Life Science, Bar Ilan University, Ramat-Gan, 52900, Israel

## Abstract

**Background:**

The adaptive immune system is based on selected populations of molecularly distinct individual B and T cell clones. However, it has not been possible to characterize these clones in a comprehensive and informatics manner to date; attempts have been limited by the number of cells in the adaptive immune system and an inability to quantify them. Recently, using the Zebrafish (ZF) *Danio rerio *as a model organism and parallel sequencing as the quantifying technology, Weinstein et al. overcame this major hurdle and quantified the entire heavy chain B-cell repertoire in ZF. Here, we present a novel network analysis of the data from the Weinstein group, providing new insights into the network structure of the B-cell repertoire.

**Results:**

Using a collection of computational methods, the IgM sequences from 14 fish were analyzed. This analysis demonstrated that the B-cell repertoire of the ZF is structured along similar lines to those previously detected in limited parts of the human B-cell immune system. The analysis confirms the validity of the global data and the evolutionary placement of the ZF based on known sequence motifs. Recombination events in the repertoire were quantified, and demonstrated a lack of shared recombined V, J groups across fish. Nevertheless, it was demonstrated that a similar network architecture is shared among fish. However, the network analysis identified two distinct populations within the group; these findings are compatible with the occurrence of an immune response in a subset of the fish. The emerging connectivity network was demonstrated and quantified, and mutation drifts within the groups were characterized. Dissection of sequence data revealed common network features of the B-cell repertoire as well as individual differences.

**Conclusion:**

The ZF B-cell repertoire reveals an underlying order that is compatible with self-organization representing every portion of the sequence-based network. This pattern varies in individual specimens, perhaps as a response to an immune challenge. However, a sequence-non-specific network that maintains a common architecture of sequence diversity was detected.

The common feature among different individuals can be captured by the network architecture and characteristics, rather than specific clones. We believe that further study of the dynamics of this network could provide insight into modes of operation of the immune system.

## Background

The immune system is a remarkably adaptive defense and maintenance system that has evolved in vertebrates to protect against invading pathogenic microorganisms and to maintain homeostasis. The immune system has two arms: the innate arm, which is activated by innate ligands, and the adaptive arm, which includes T cells and B cells that recognize antigens via their specific antigen receptors (TCR and BCR) [1,2].

B cells, a component of the adaptive immune system, mature within the bone marrow; when they exit to the periphery as naive B cells they express a unique antigen-binding receptor, immunoglobulin (Ig), on their membrane. When activated by the antigen specific to its membrane-bound antibody, a B cell proliferates and differentiates to generate plasma cells that secrete Ig molecules, and also memory cells [1,3,4].

B-cell maturation depends on rearrangement of the Ig in a process known as V (D) J rearrangement. By randomly choosing V, D and J genes among many alleles, the recombination provides a variety of antigen sequences. The process is highly conserved in jawed vertebrates [5,6]. The whole collection of various, rearranged immune receptors is known as the B-cell repertoire.

Additional variability within the B-cell repertoire arises from somatic hypermutation (SHM) - a recombination process that occurs in germinal centers in which the recombined immunoglobulin undergoes error-prone replication during an *in vivo *selection process. These mutations are several orders of magnitude more frequent than in genes encoding other proteins [7-11]. Several mutations yield amino acid substitutions that improve antigen binding by increasing the antigen affinity and diversity [12].

The Zebrafish (ZF), *Danio rerio*, offers unique opportunities for studying the ontogenetic development of the immune system. A great advantage in studying this organism is the optical transparency of ZF during early development and the fact that it shares many orthologous genes with mouse and man (e.g., rag1 and rag2). This gives the species considerable relevance over other traditional developmental models [13-15]. The ZF immune system has approximately 300,000 antibody-producing cells. This is a small number compared to an estimated 10^12 ^cells in humans. Therefore, ZF is an excellent model organism for global quantitative analysis of the immune system, including the B-cell repertoire.

Technically, questions concerning the B cell repertoire have been limited by: (1) the scale of the system, with more than 10^12 ^cells in humans and a similar order of magnitude in mice; (2) an inability to measure diversity within the whole population of B cells. The scale of the system is solved by using ZF as a model organism, with 10^5 ^B cells generating an immune system of comparable complexity. Quantifying diversity is currently approachable using platforms of massive parallel sequencing. While such systems cannot sequence recombined regions (~200 bp in length) from 10^12 ^cells within a reasonable time frame, they are capable of sequencing recombined regions from the approximate 10^5 ^cells of ZF.

Recently, Weinstein et al. [16] constructed the sequence reads of 640 million bases of zebrafish antibody heavy chain cDNA from 14 zebrafish in four families, and the data are publicly available. The authors characterized and analyzed the antibody repertoire of zebrafish by analyzing complementary determining region 3 (CDR3) of the heavy chain.

The concept of network architecture has changed the perspective on biological phenomena recently [17-19]. Research has demonstrated the importance of network architecture in terms of phenotype classification, emergence and function (for a recent review, see [20]). Such research suggests, for example, that the topological prominence of a protein in a protein interaction is a good predictor of its biological importance. Network architecture analysis provides the tools to study structural properties in networks. One important method is centrality analysis. Such analysis ranks vertices in a network according to their connectivity within a network structure [18,21,22]. Centrality indices of a network include the Degree of each vertex (the number of physical connections a vertex has) and the Betweenness of a vertex which measure the number of shortest paths that the vertex lies on (high Betweenness signifies this vertex as one that resides on several short paths). Betweenness of a node is the proportion of shortest paths among all pairs of reachable nodes that go through the node; Betweenness measures the centrality of a node in the global network structure [23].

Protein-Protein interaction networks (PPI) becoming a key strategy for uncovering the inner mechanism of a cell, these networks systematically determine both the potential and actual protein interactions in selected model organisms, such as the S. cerevisiae. PPI networks identify every node with a specific protein and the edges stands for identified direct physical interaction. The protein interactions and its position in the network are likely to correlate with the protein functional properties. The nodes themselves possess characteristics that carry important information about their role in the system, (e.g highly connected nodes tend to be more essential) [23-26].

In the present study, the dataset of Weinstein et al.[16] was analyzed to characterize the whole recombined sequence, and to investigate V (D) J distribution among all 14 fish and the mutations that occur within the sequences. In addition, the Weinstein data were extended to characterize the connectivity network of the ZF B-cell repertoire and study mutation drifts as multiple nodes within the groups. Unlike PPI networks here every node represent a different sequence in the repertoire and edges represents mutations or indels (insertion or deletion) that may occurred. This form of network-based interpretation facilitates the identification of unique sequences versus groups of sequences and indicates their centrality in the network. Using these computational tools that facilitate the dissection of sequence data, the fundamental repertoire architecture was examined to investigate how the repertoire varies among individuals and the common network features that dictate the B-cell repertoire. Further, we report that in the dataset of Weinstein et al. there are two distinct immune behaviors, which have not been identified in previous analyses [16,27]. We hypothesize that these distinct immune behaviors are immune responses that may be the result of external perturbations such as a yet unknown pathogenic response.

## Results and Discussions

### Network Analysis

The Ig network that emerged from the Ig sequences in each fish was analyzed. In addition, to highlight the specific choices in the selection process, the observed network was compared with an artificial, randomly-based network. The Random network algorithm randomly chose a combination of three germline sequences (V, D and J) in order to build a "naive" sequence, then the algorithm randomly put point mutations, deletions and insertions into every sequence. The random network does not represent a population of B cell but the lowest level of organization, one with no bias toward any specific germline combination or mutation.

**Figure 1:**
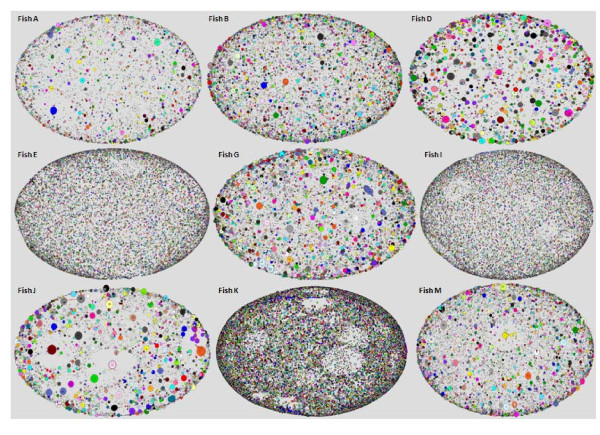
**Ig networks were divided into groups according to the repertoire profile**. Poorly connected networks that were constructed from nine fish. Every node represents a sequence and the node size refers to the number of identical sequences; node color refers to the connected component that the node is a part of (every connected component has a different color).

**Figure 2:**
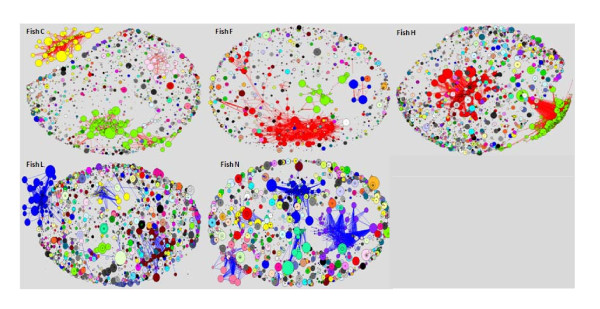
**Highly connected Ig networks from five fish that showed similar repertoire profile**. Every node represents a sequence and the node size refers to the number of identical sequences; node color refers to the connected component that the node is a part of (every connected component has a different color).

**Figure 3:**
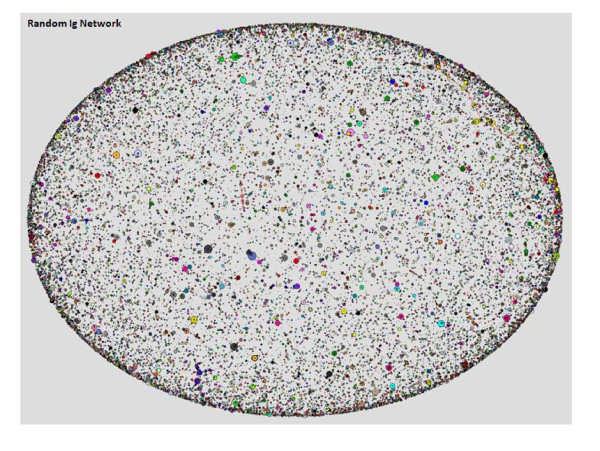
**Random Ig Network that was constructed from random perturbations over the germline sequences**. This network contains 50,000 sequences that were fitted into 43,491 vertices. Maximum vertex size is 18 and maximum Degree in the network is 16. Every node represents a sequence and the node size refers to the number of identical sequences; node color refers to the connected component that the node is a part of (every connected component has a different color).

The network analysis separated a group of five fish with highly developed networks from the remaining nine fish that demonstrated more naïve, less differentiated networks. Figure 1 and 2 illustrates all networks that emerged from the individual fish classified to two groups according to their specific network behavior and organization, and figure 3 the random network. It is reasonable to suppose that the more differentiated networks reflect an immune response, while the less connected networks reflect a more stable state.

A metric evaluation of the Ig network architecture demonstrated major differences between the groups.

Each vertex in the network refers to a different sequence and the size of the vertex represents the number of identical sequences. A red edge indicates a point mutation between the two sequences, a blue edge indicates one deletion and a green edge represents deletion and mutation. Each component is marked by a distinct color, so that the node color ascribes the node to a specific connected component of the network.

Fish in the highly connected group exhibit large clusters of components. Such clusters may be the network equivalent of an immune system response to a foreign antigen. The rationale behind this categorization of inflamed vs. naive fish is that the basis for network behavior is similarity between clones, or clonal expansion. The components are highly connected, and the vertices in these clusters are bigger than those of relative networks. In the poorly connected group of fish (such as fish D) the same distribution of all sequences is observed and the highly connected, massive components are noticeably absent from such networks. We would expect to observe 975 (39V × 5D × 5J) connected clusters, representing all the possible V (D) J combinations. However, hundreds of thousands of components were identified. Therefore, the mutation process is such that once sequences have been generated, the network algorithm cannot connect them to one another in a single step.

**Figure 4:**
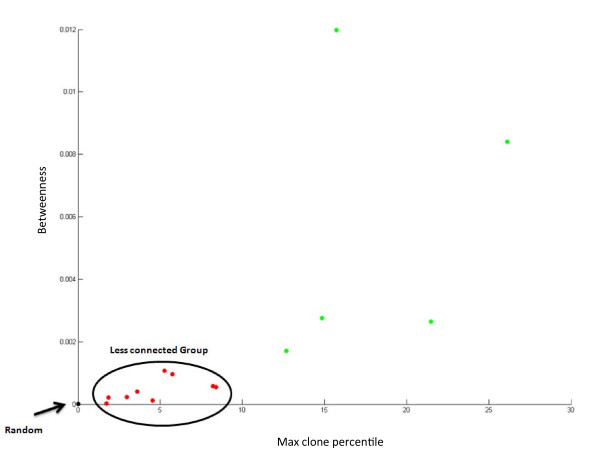
**Centrality indices differ among three populations**. Differences between random, poorly connected and highly connected networks, in terms of Betweenness VS the percentile of the biggest clone, compared with the number of sequences in the fish. This index measures the biggest clone in every fish (percentage) in order to compare fish with different numbers of sequences. Big clones will not normally characterize a "stable" state.

**Figure 5:**
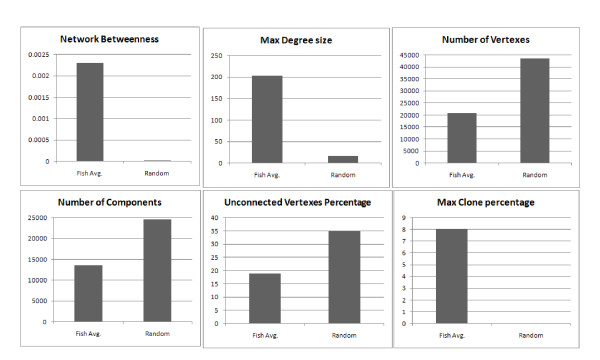
**Comparison between centrality indices in the real Ig networks and the random one**.

In addition, the random network displays a very different network structure, with all indices differing markedly from networks observed in fish Ig sequences. Differences were observed in vertex degree, the number of identical sequences and the number of unconnected vertices. Figure 4 presents the three distinct populations and figure 5 demonstrates differences between the random network indices and the average indices across all 14 fish. This contrast demonstrates how different the self-organized architecture of the immune network is from a randomly-based architecture. While the sequences themselves may portray a random-like distribution with no preferred sequences across individuals, the network reveals a shared blueprint. This kind of visualization reveals three levels of architecture, from the most naive unbiased network to a highly connected network, which may unfold the specific processes of the immune response.

As described previously, networks can be assessed by studying and comparing their centrality indices, and in the present study we examined the Degree and Betweenness of the vertex in every network and the size of the vertex. This analysis demonstrated that poorly connected fish have vertices that contain a maximum of 10% of their sequences, while highly connected fish have vertices that contain 50% of their sequences. Furthermore, the vertices in connected fish were of much higher degree than in poorly connected fish, as were levels of network Betweenness centrality, indicating several connected clones that could be the result of an immune response in those fish.

### V/J segment analysis

The Ig sequences from the original paper by Weinstein et al. were obtained from 14 fish, with each family being raised in separate aquaria and having normal interactions with the environment including the development of natural internal flora [16]. V and J segments from all 14 fish were analyzed, and germline sequences were matched with sequence reads to demonstrate their distribution among individual fish.

**Figure 6:**
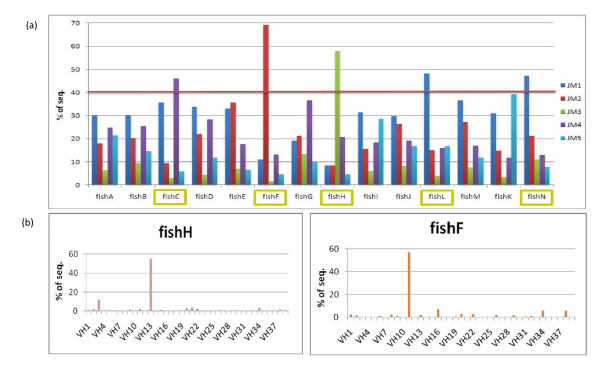
**VJ genes distribution across fish shows similar architecture as the networks**. (A) J gene distribution across fish. Five fish demonstrate that more than 40% of their sequences are sourced from a specific gene; the same fish had highly connected network architecture. (B) The same fish demonstrate a similar behavior in their choices of specific V genes, as shown for fish H and fish F. Fish with highly connected network architecture had distinct germline representation.

**Figure 7:**
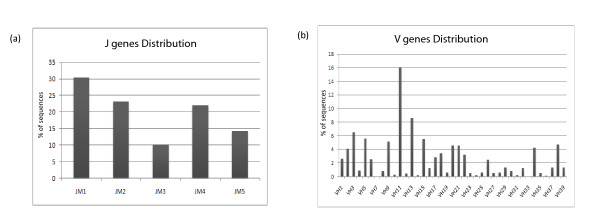
**Average gene distribution**. (A) The J gene average distribution across all 14 fish. (B) The V genes average distribution across all 14 fish. Average distribution was adjusted to the number of sequences in every fish by calculating the percentile; the figure demonstrates how VJ sequences are used across the entire population.

The analysis suggested that the 14 ZF could be separated into two distinct groups. In five of them, the same five that presented with highly connected network architecture, at least 40% of the Ig sequences could be attributed to single germline combinations, in both V and J genes. Figure 6 demonstrates the distribution of J gene alleles among the fish; figure 7 demonstrates the average distribution of V and J genes. The five fish marked by yellow squares in figure 6(A) represent fish with over 40% specific V or J sequences. This skewing of the B-cell clonal repertoire cannot be explained by errors in PCR amplification because the over-expression accounts for a specific V or J germline gene and not for a specific sequence. PCR amplification errors produce a bias towards specific over-expressed sequences but not to a specific germline.

**Figure 8:**
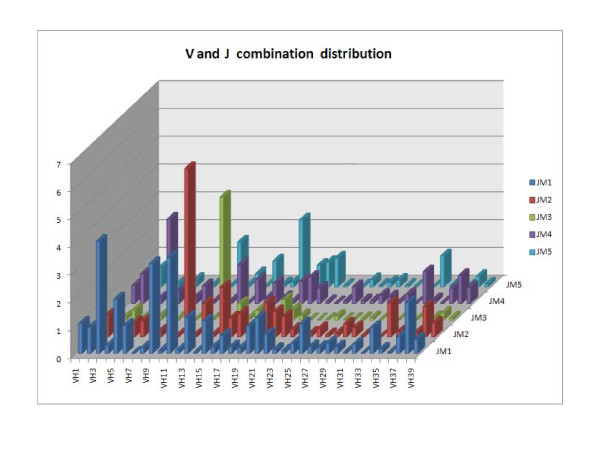
**The distribution of all possible V and J combination in all 14 fishes, resulting in 195 possibilities**.

To investigate whether specific clones dominate the repertoire, VJ recombination was quantified across the fish. Specific combinations accounted for up to 6% of all sequences (Figure 8). Perhaps more importantly, there was a scarcity of rare combinations, accounting for less than 0.001% of all sequences (i.e. combinations found in fewer than 10 sequences).

### Mutation Analysis

One of the hallmarks of the adaptive immune system is its ability to recognize large numbers of antigens. B-cell repertoire diversity is generated by two distinct processes that modify the Ig sequence.

The primary repertoire is generated in the bone marrow by a process of V (D) J recombination, with further diversification via two pathways: (1) somatic hyper-mutation (SHM), a process that introduces a high frequency of point mutations into the variable region gene of the Ig; (2) relatively rare insertions and deletions of nucleotides. Recent studies have demonstrated that the molecular processes of SHM and class-switch recombination (CSR) require the activity of activation-induced cytidine deaminase (AID), which removes C to form a U nucleotide on the mRNA sequence. This change activates proteins that mediate SHM and CSR [28-30].

**Figure 9:**
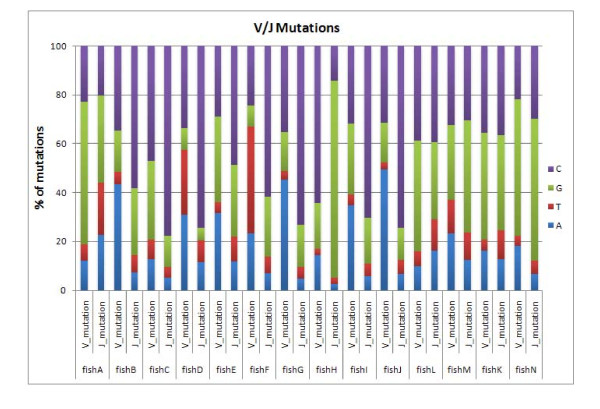
**Percentage of mutations in the V and J genes in all 14 fishes; 46% of mutations from C, 36% of mutations from G, 14% of mutations from A and 4% mutations from T**.

By aligning the sequences to their germline, the type of mutations that occurred could be quantified. This kind of analysis in the V and J segments revealed that 46% of all mutations originated from C residues, as shown in Figure 9; this finding is consistent with the AID mechanism [31].

**Figure 10:**
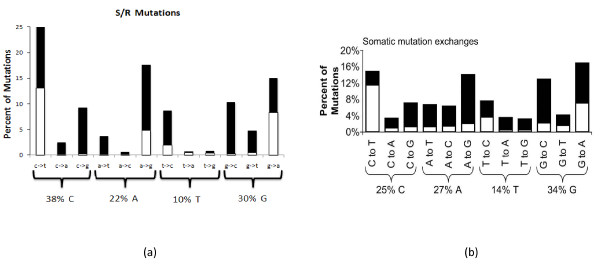
**Silent (white areas of bars) vs. replacement (black areas of bars) mutations**. (A) Percentage of mutations in all 14 fish as constructed from the data. (B) Percentage of somatic mutation exchanges in humans as presented in [28].

Silent (synonymous) mutations do not result in the replacement of amino acids, as opposed to non-synonymous mutations, which change the sequence of amino acids in the resulting polypeptide. Silent vs. replacement mutations in the V and J genes were investigated (Figure 10A). The results were compared with published data concerning V genes in humans [28], which demonstrate that somatically mutated human IgV_H _genes accumulate C-to-T base exchanges that are predominantly silent. The ZF sequences manifested the same three major replacements (peaks in Figure 10a) as in humans (C-to-T, A-to-G, G-to-A), and more silent mutations in C-to-T and in G-to-A. It was reasoned that such a bias would reduce the tendency of somatic mutations to occur in C compared to A nucleotides. A recent study has demonstrated that the over-expression of codons with silent mutations in *C*/*T *is not merely a mechanism to reduce the propensity for change in *C *but is balanced by the use of more changeable codons and AAs [32].

ZF share several orthologous genes with mouse and human, which gives this species considerable relevance over other traditional developmental models [13]. Moreover, previous works have identified several regions of the ZF and human genome that encode the same or similar genes [33-35]. In addition, the results presented herein reinforce the similarity between these two species at the level of the mutation process and base specificity.

### Sequence Motifs

**Figure 11:**
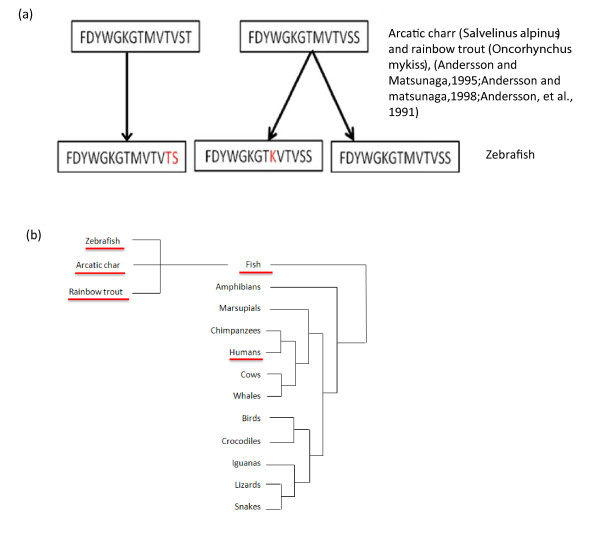
**Motifs found in ZF and the proximity to humans**. (A) Motif in the zebrafish sequences identified in this work compared to sequence motifs that were previously identified in Arctic charr (*Salvelinus alpinus*) and rainbow trout (*Oncorhynchus mykiss*) [1-3]. (B) Phylogenetic tree representing the proximity between humans and fish (zebrafish, arcatic char, rainbow trout).

Sequences motifs are a collection of amino acid residues found in various organisms, and shared sequence motifs can imply an evolutionary link between organisms. Certain amino acid residues are unique to the VH genes of various vertebrate species [4,36,37], and considered markers for the sequence homogenization process in Ig gene evolution. The recurring sequences FDYWGKGTMVTVST and FDYWGKGTMVTVSS were previously identified in two teleost fish species, Arctic charr (*Salvelinus alpinus*) and rainbow trout (*Oncorhynchus mykiss*) [4,36,38], which are separated from their common ancestor by 12-20 million years. The same motif, FDYWGKGTMVTVSS, was evident in the zebrafish DH-JH region and the sequence motif **FDYWGKGTMVTVST **was absent (Figure 11). Two new motifs, probably resulting from favorable mutations that occurred during evolution, were identified; from both motifs: **FDYWGKGTMVTVTS **and **FDYWGKGTKVTVSS**.

In addition to the DH-JH region motifs identified, the specific motif **EDTAVYYCAR **was present in the VH region; part of this motif (AVYYCAR) was previously identified in the TCR cDNA V region of Horn shark (*Heterodontus francisci *) [39]. These findings support the idea that the Ig heavy chain in teleosts is evolutionarily stable.

## Conclusion

Comprehensive analysis of the first sequenced whole organism Ig repertoire, as provided by Weinstein et al., resulted in the following conclusions:

• By comparing somatic mutation exchange across silent and replacement mutations, a remarkable similarity between human and ZF was evident, a similarity that places the ZF within a proper context as a relevant model for studying selection mechanisms.

• The ZF evolutionary context was investigated by comparing sequence motifs identified in ZF to evolutionary adjacent fish species.

• VJ combination analysis across individuals was carried out in order to investigate how recombined VJ regions are used in different individuals. This analysis revealed that specific portions of the repertoire are utilized by the recombination mechanisms, while others are clearly absent (figure 8).

• Mora et al. demonstrated that the data from Weinstein et al. prove that "antibody diversity is not limited by the sequences encoded in the genome" [40]. Furthermore, the authors demonstrated an intriguing set of properties that they derived from the data. However, the original paper by Weinstein et al. and the paper by Mora et al. do not demonstrate the clear stratification of the group of fish, as presented in this study. This research has demonstrated that there are two distinct groups of fish. One group presents a uniform use of VJ recombination and a connected, uniform-like network of Ig molecules, while the other presents a much higher frequency of a subset of VJ recombination and a highly connected sub-network of Ig molecules.

We hypothesize that these two groups represent two distinct populations. The group that represents a random-like pattern of behavior is composed of fish with a basal immune response, while the second set of fish represents those with a more complex immune response that may be the result of external perturbations. The network transformation performed over sequences demonstrates this visually by highlighting the special connectivity patterns, and quantifiably by using network architecture metrics that demonstrate major differences between the two groups.

The mechanisms that determine the connections made by individual sequences across the network, and the meaning of these network connections, are the subject of future work. The temporal behavior of the network during an immune response will undoubtedly shed light on the specific choices made among clones.

The shape of the immunological repertoire is at the heart of our understanding of an immune response, self-non-self discrimination, autoimmunity and immune modulation. As current technologies facilitate organism-wide measurements, a better understanding of this fundamental system is tied to our ability to influence the immunological repertoire in a clinically related manner.

## Methods

All sequences were obtained from supporting online information from the report published by Weinstein et al. [16]. A total of 14 fish and approximately one million sequences were used. Germline sequences were obtained from Danilova et al. [41].

The Zebrafish heavy chain has 39 V genes, 5 D genes and 5 J genes: overall, there are 975 (39 × 5 × 5) optional combinations of V (D) J sequences [16]. Here, using BLAST [42] alignment, V and J germline sequences were matched. From a sequence perspective, the five D genes were very close. Therefore, D gene alignment was not used as an indicator and the D region was defined for analyses as the segment between the identifiable parts of V and J [27].

All sequences with a BLAST E-value higher than 10^-8 ^were removed from the analysis owing to insufficient fit to either V or J (overall, less than 0.5% sequences were extracted). Results presented have been normalized across individual fish.

Mutation analysis was carried out by aligning each sequence to its germline and tallying base substitutions, deletions and insertions in the sequence. Once all mutations were determined, the ORF was identified and the type of mutation was tagged as silent or replacement, thus tallying the amount of silent and replacement mutations and their frequencies within the four nucleotides. The results were compared with the analysis previously carried out on humans [28]. The nucleotide sequences were translated into amino acid sequences in order to be analyzed and to allow the search for specific motifs that are consistent in the majority of sequences. This analysis revealed a pattern that was previously found in other vertebrates.

Network analysis was carried out using Pajek [43], a program for network layout and analyses. Each vertex in the network represents a different sequence. The size of the vertex is comparable to the number of sequences matching a specific vertex perfectly. The distance between a pair of connected nodes in the network is one mutation (red edge), one deletion (blue edge) or mutation and deletion (green edge). Nodes in every cluster are colored differently, such that each cluster represents a connected component inside the whole network. In other words, in every component there is a path connecting every two vertices. Network centrality indices, which are a key measurement in assessing any network, were calculated using Pajek and are presented in the results section.

## Authors' contributions

RBH and SE designed, analyzed and wrote the paper. All authors read and approved the final manuscript.
